# Causal Structure of Brain Physiology after Brain Injury from Subarachnoid Hemorrhage

**DOI:** 10.1371/journal.pone.0149878

**Published:** 2016-04-28

**Authors:** Jan Claassen, Shah Atiqur Rahman, Yuxiao Huang, Hans-Peter Frey, J. Michael Schmidt, David Albers, Cristina Maria Falo, Soojin Park, Sachin Agarwal, E. Sander Connolly, Samantha Kleinberg

**Affiliations:** 1 Division of Critical Care Neurology, Department of Neurology, Columbia University, New York, NY, United States of America; 2 Computer Science Department, Stevens Institute of Technology, Hoboken, NJ, United States of America; 3 Department of Biomedical Informatics, Columbia University, New York, NY, United States of America; 4 Department of Neurosurgery, Columbia University, New York, NY, United States of America; University of Pennsylvania, UNITED STATES

## Abstract

High frequency physiologic data are routinely generated for intensive care patients. While massive amounts of data make it difficult for clinicians to extract meaningful signals, these data could provide insight into the state of critically ill patients and guide interventions. We develop uniquely customized computational methods to uncover the causal structure within systemic and brain physiologic measures recorded in a neurological intensive care unit after subarachnoid hemorrhage. While the data have many missing values, poor signal-to-noise ratio, and are composed from a heterogeneous patient population, our advanced imputation and causal inference techniques enable physiologic models to be learned for individuals. Our analyses confirm that complex physiologic relationships including demand and supply of oxygen underlie brain oxygen measurements and that mechanisms for brain swelling early after injury may differ from those that develop in a delayed fashion. These inference methods will enable wider use of ICU data to understand patient physiology.

## Introduction

Novel therapeutic interventions are desperately needed for acute brain injuries, which affect large parts of the population worldwide and are associated with poor long-term outcome.[1] Interventions are traditionally developed on animal models or simulated data, but translation to humans often fails, as these models inaccurately represent human pathophysiology. Clinical trials are usually built upon observational and associative data in the pathologic state but rarely take the complex underlying biological dependencies into account. Clinical trials in acute brain injury take a reductionist interpretation of biological systems, often ignoring complex underlying physiological dependencies. Built upon observational and associative data in the pathologic state, they have failed to show a benefit. Such clinical trials for acute brain injury include high oxygen therapy after traumatic brain injury (TBI),[2] tight glucose control for critically ill brain injured patients,[3] and erythropoietin administration and blood transfusions for TBI patients.[4]

Understanding causal inferences amongst biological signals recorded shortly after injury would allow us to better determine the extent to which experimental data replicates the real world scenario. Clinical data are obtained at a time when interventions have the highest chance of changing the disease course but are challenging to analyze due to incomplete data sets, poor signal-to-noise ratios, non-stationarity, hidden dependencies, and correlations on different time scales. Causal inference methods have been successfully applied in neuroscience[5] to understand sleep[6] and seizure dynamics,[7] but have yet to find success in clinical settings encountered acutely after brain injury. Computational approaches that have been successful in other areas, however, often do not address the challenges of clinically generated biological data and there is a need for specialized methods to address this.

Spontaneous subarachnoid hemorrhage (SAH) is an acute brain injury that frequently leaves patients comatose and can lead to poor outcomes.[8, 9] Comatose patients may appear like a black box to clinicians for whom it is difficult to determine if their patient is improving or requires changes to their treatment. ICU patients generate hundreds of thousands of physiologic data points during their ICU course representing a number of different physiologic and pathophysiologic processes. In this study we hypothesize that physiologically meaningful causal inferences can be identified from high frequency physiologic data recorded from acute SAH patients.

## Materials and Methods

### Study population

We studied all poor-grade aneurysmal SAH-patients in coma (defined as those with a Glasgow Coma Score of 8 or below) admitted to Columbia University Medical Center between June 2006 and October 2013 that underwent invasive brain multimodality monitoring. The diagnosis of SAH was established by computed tomography (CT) or xanthochromia of the cerebrospinal fluid if the CT was negative. Patients were not enrolled if any of the following were met [1] age <18 years old, [2] pregnant, or [3] patients or families did not want to participate in the study. Medical and surgical treatment followed guidelines by the American Heart Association and existing management protocols at most large medical centers.[10, 11]

### Ethics statement

Data were collected as part of an ongoing prospective database approved by the local Institutional Review Board. Columbia University Medical Center IRB (study identification number: AAAL4106) provided approval for all data presented in the current submission. All research activities have been conducted according to the principles expressed in the Declaration of Helsinki and in compliance with all local, state, federal, and IRB mandated policies related to human subjects research. Written consent was obtained when a patient and/or their legally authorized representative, health proxy or surrogate was available to provide written consent. As such the CUMC IRB approved a waiver of documentation of consent as this is a no more than minimal risk observational outcomes study. For patients that we were able to obtain a written signed consent form, the CUMC IRB approved document was presented to the patient and/or their surrogate (as defined below), upon obtaining consent. For patients who were not able to provide a written consent, the CUMC IRB approved a Information Sheet that was provided to the patient. If a surrogate was found then the study team sought written consent, or when the patient was able to provide informed consent, they were presented with the option of continuing participation, not participating in long term follow up but allowing deidentified collection of their hospitalization data, or total removal from the study.

### Multimodality monitoring

According to our protocol[12, 13] invasive neuromonitoring includes measurements of intracranial pressure (Integra Neurosciences Inc, Plainsborough, NJ), interstitial cerebral microdialysis (CMA-70 microdialysis catheter^TM^ [20 kDa pores], analyzed for lactate, pyruvate, and glucose using the CMA-600^TM^, CMA Inc, Stockholm, Sweden; metabolic crisis was defined as lactate pyruvate ratio [LPR] > 40 and brain glucose < 0.7mmol/L), partial brain tissue oxygenation (PbtO2) and brain temperature (using a flexible polarographic Licox Clark-type probe; LICOX^TM^, Integra Neurosciences Inc, Kiel, Germany), regional cerebral blood flow (Bowman Perfusion Monitor^TM^, Hemedex Inc, Cambridge, MA). Physiologic measurements were categorized into normal ranges based on recommendations form the literature or consensus between experienced neurointensevists and neurosurgeons (see Table 1 for details).

**Table 1:** Systemic and brain physiologic parameters.

	Normal Range*	Reference for normal range	Monitoring device, measuring technique
**Systemic parameters**			
TMP, body temperature [°C]	36.5–38.5	[14]	Bladder temperature probe, Bardex^TM^, Bard Medical
RR, respiratory rate [cycles per minute]	10–18	Expert Consensus	840-Puritan Bennett^TM^, Covidien
MV, minute ventilation [cycles per minute]	5–8	Expert Consensus	840-Puritan Bennett^TM^, Covidien
CO2EX, end tidal carbon dioxide [mmHg]	35–45	[15]	Infrared capnometer, Respironics^TM^, Philips
SPO2%, oxygen saturation [%]	>89	[15]	Covidien Nellcore
HR, heart rate [beats per minute]	60–80	Expert Consensus	General Electric Solar 8000i monitors
MAP, mean arterial pressure [mmHg]	70–110	Expert Consensus	General Electric Solar 8000i monitors
CVP, central venous pressure [mmHg]	2.0–6.0	[16]	Central venous line; General Electric Solar 8000i monitors
CI, cardiac index	2.4–4.0	[16]	Pulse contour analysis (Vigileo, Edwards Life Science; PICCO, Pulsion, Phillips)
SVV, stroke volume variation [%]	< = 12	[16]	Pulse contour analysis (Vigileo, Edwards Life Science; PICCO, Pulsion, Phillips)
ELWI, extravascular lung water index [ml/kg]	3–7	[16]	Pulse contour analysis (Vigileo, Edwards Life Science; PICCO, Pulsion, Phillips)
GEDI, global enddiastolic index [ml/m^2^]	680–800	[16]	Pulse contour analysis (Vigileo, Edwards Life Science; PICCO, Pulsion, Phillips)
**Brain physiology**			
ICP, intracranial pressure [mmHg]	<20	[17, 18]	Integra Neurosciences^TM^
CPP, cerebral perfusion pressure [mmHg]	60–90	[15, 18]	Integra Neurosciences^TM^
pbtO2, partial brain tissue oxygenation [mmHg]	>15	[15]	LICOX^TM^, Integra Neurosciences
rCBF, regional cerebral blood flow [ml/100g/min]	>35	[19]	Bowman Perfusion Monitor^TM^, Hemedex
TW%, brain water content [%]	n.a.	[19, 20]	Bowman Perfusion Monitor^TM^, Hemedex
BrT, brain temperature [°C]	36.5–37.5	[19]	Bowman Perfusion Monitor^TM^, Hemedex
SjVo2, jugular bulb oxygenation [%]	50–75	[15]	Continuous Fiberoptic Central Venous Oximetry, Edwards Lifescience
Interstitial lactate [mmol/L]	2.7–3.3	[21]	CMA-70 microdialysis^TM^, MDialysis
Interstitial pyruvate [μmol/L]	138.6–1.1.4	[21]	CMA-70 microdialysis^TM^, MDialysis
Interstitial glucose [mmol/L]	1.9–2.3	[21]	CMA-70 microdialysis^TM^, MDialysis
Interstitial lactate pyruvate ratio	<40	[21]	CMA-70 microdialysis^TM^, MDialysis

* The normal range for several of these is controversial. For the purposes of this study we chose previously published recommendations as referenced above.

### Data collection

Physiologic data was acquired using a high-resolution acquisition system (BedmasterEX^TM^; Excel Medical Electronics Inc) from General Electric Solar 8000i monitors and inserted into a Microsoft SQL database. Additionally we collected clinical variables including demographics, disease specific variables (i.e., aneurysm size), laboratory values, neurological examination findings, medications, hospital complications, and three months functional outcome data (modified Rankin Scale) as part of an ongoing, prospective observational SAH outcomes study.

### Data preparation

To account for varying time resolutions of the collected data we first created minute averages for all measurements. However, microdialysis measures were not available more than every 30 minutes and were normally approximately an hour apart. We removed physiologically implausible values [outliers) following a previously described approach.[13] We calculated the cerebral perfusion pressure (CPP) by subtracting the minute averages of intracranial pressure (ICP) from the mean arterial pressure (MAP). Total brain water content (TW%) is based on thermal conductivity measurements made through the calibration process of the Hemedex^TM^ monitor applying previously described methodology.[20]

Clinically recorded physiological data may have gaps due to device malfunction or loss of connectivity between the recording and data storage location. After synchronizing the data, and averaging each minute, missing values were imputed using the FLk-NN method (https://github.com/kleinberg-larb/FLK-NN).[22] This approach combines two methods–imputation based on k-nearest neighbors with lagged correlations (Lk-NN) and the fast Fourier transform (F) to address two types of missingness–one that depends on the variable itself and the other that depends on other variables. Key parameters are the number of neighbors for k-NN, set as 5 (which gave the highest accuracy in our preliminary tests with simulated missingness) and the number of time lags (which is set as 1 hour, as this should capture most biologically significant relationships). One additional parameter, p, is needed, which is the number of lags for each relationships, which was set as 3 to capture a range of time lags without being computationally prohibitive. Finally, variables for causes were binned according to clinically accepted ranges.

### Causal Inference

Instead of testing specific hypotheses, we aim to infer the causal structure between the measured variables in a data driven way. Thus we tested for causal relationships between all measured variables and all other variables at a series of timescales (5 minute windows up to one hour). Because of the heterogeneity of ICU patients and the amount of data collected on each, inference was done for each patient separately (so that 98 separate models were learned). The inference method infers causal relationships and their timing from time-series data, by evaluating how much of an effect’s value a cause explains, relative to other potential causes.[23] This method, implemented in Common Lisp, has been validated in a number of domains (finance, biology) and has previously been applied to electronic health record data, where it found causes of congestive heart failure at multiple timescales.[24] The output is a set of values for ϵ_avg_, the causal significance for each relationship (a cause, effect, and time window), which represents the average impact of the cause on the effect’s value when holding fixed other potential causes. However a threshold must be chosen to determine which values of this measure are statistically significant. Significant relationships for each patient were determined using the causal significance scores and a p-value cutoff of 0.05. Since models were inferred for individuals and each is a separate family for the purposes of hypothesis testing, and the causal significance is only calculated for causes that seem to raise the probability of their effects, there were not a large number of causes tested in each step and thus we did not control for multiple hypothesis testing as we normally would. We explored causal relationships during two predefined time periods or phases: days 0 to 3 after bleed; and days 4 to 7 after bleed.[25, 26]

### Phenotyping

We performed k-means clustering to identify specific phenotypes of patients that did have causal relationships identified. Clustering was performed using “kmeans” implemented in R statistical language (v. 3.1.3) using parameters that are known to affect hospital course, such as severity of disease, age, or development of cerebral edema, as well as outcome parameters.[27] The optimal number of clusters was determined using the gap method 22 based on 500 reference data sets.

## Results

### Patients

**Fig 1:**
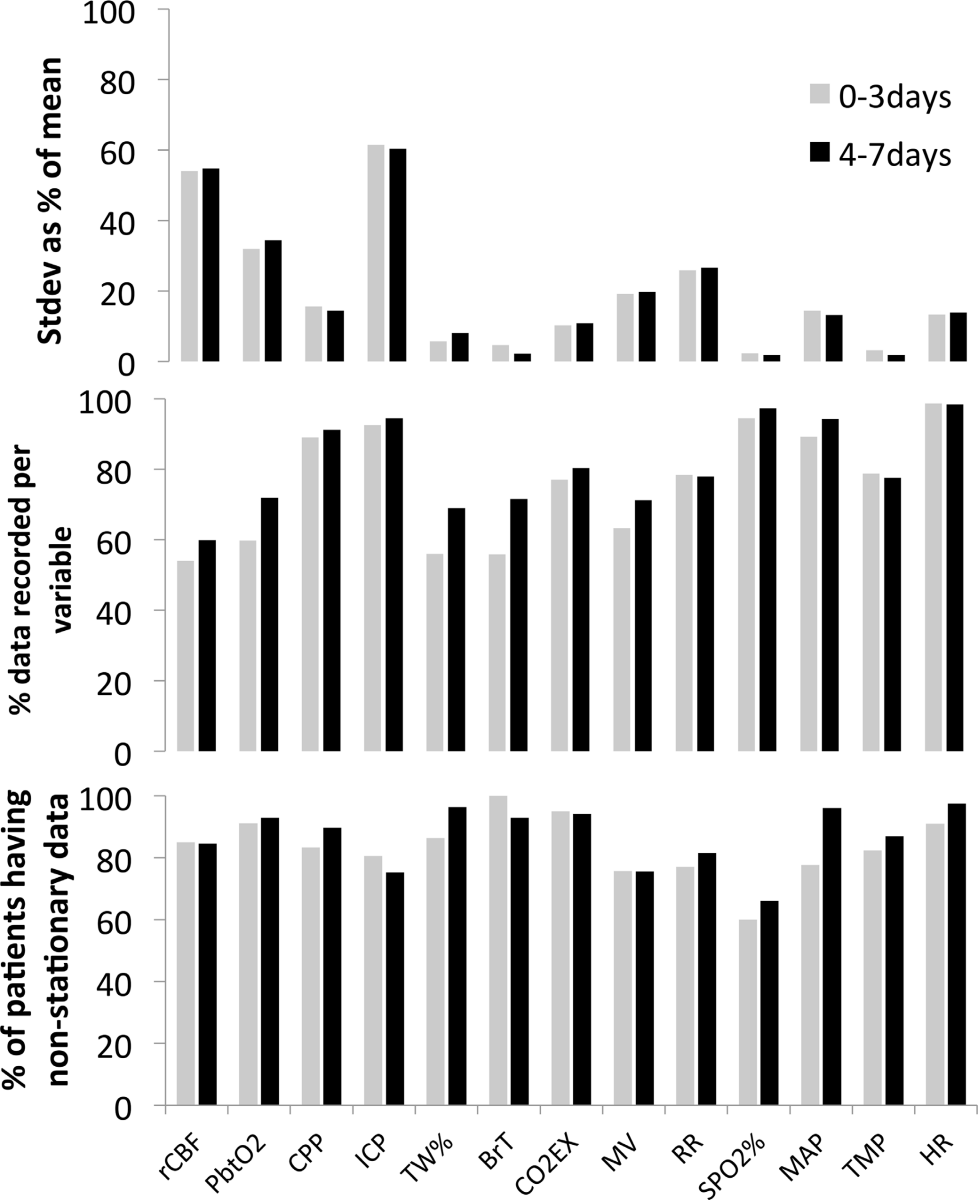
Data characteristics of the collected physiologic measures at two different time periods following brain hemorrhage (grey bars 0 to 3 days, black bars 4 to 7 days after SAH). Standard deviation represented as percent of the mean (top panel), average percent of time that a specific variable was available from the overall monitoring time (middle), and KPSS test illustrating the percent of patients that have non-stationary data for a variable (bottom panel; calculated as the ratio of the number of patients that have p-val < = 0.01) divided by total number of patients that have the variable.

We identified 98 consecutive SAH patients that underwent multimodality monitoring (MMM). All patients were intubated and blood pressure was tightly controlled requiring vasopressors in 91% (N = 89) and antihypertensives in 77% (N = 75) of the cohort (Table 2). Monitoring was started on post SAH bleed day 3.0+/-2.6. During the initial phase (post bleed days 0 to 3) and secondary phase (post bleed days 4 to 7) brain monitoring data were available in 74% (N = 73) and 95% (N = 93) of patients, respectively. Low variation and high nonstationarity (using the KPSS test) were seen for total brain water content (TW%), brain temperature (BrT), oxygen saturation (SPO2), and body temperature, while regional cerebral blood flow (rCBF), partial brain tissue oxygenation (pbtO2) had fewer data points (Fig 1). Mostly present data along with high variation, and nonstationarity were observed for intracranial pressure (ICP). These data characteristics did not change in between the two time periods.

**Table 2:** Baseline features of included SAH patients with multimodality monitoring compared to SAH patients without.

	SAH patients With MMM (N = 98)	SAH patients Without MMM (N = 1556)	P-value
**Demographics**			
Age [years]	54+/-14.5	55+/-14.7	N.S.
Female	66 (67)	1043 (67)	N.S.
White	32 (33)	707 (45)	.014
**Admission findings**			
Hunt Hess score	5 (4, 5)	3 (2, 4)	< .001
APACHE 2 score	22+/-7	12+/-8	< .001
Modified Fisher Score	3 (3, 4)	3 (1, 3)	.005
IVH	76 (78)	766 (49)	< .001
Global cerebral edema	65 (66)	397 (26)	< .001
Aneurysm clipping	52 (53)	874 (56)	N.S.
**MMM devices available**			
Parenchymal ICP monitor	58 (59)	n.a.	
pbtO2	80 (82)	n.a.	
rCBF, TW%, BrT	37 (38)	n.a.	
**Hospital course**			
Any in-hospital seizure	24 (24)	99 (6)	< .001
Sepsis	18 (18)	127 (8)	.001
Delayed cerebral ischemia	31 (32)	292 (19)	.002
**Functional outcome at 3 months**			
Modified Rankin score	4 (3, 6)	2 (1, 5)	< .001

Data are shown as number (%), mean +/- standard deviation, or median (IQR 25%, IQR 75%).

**Fig 2:**
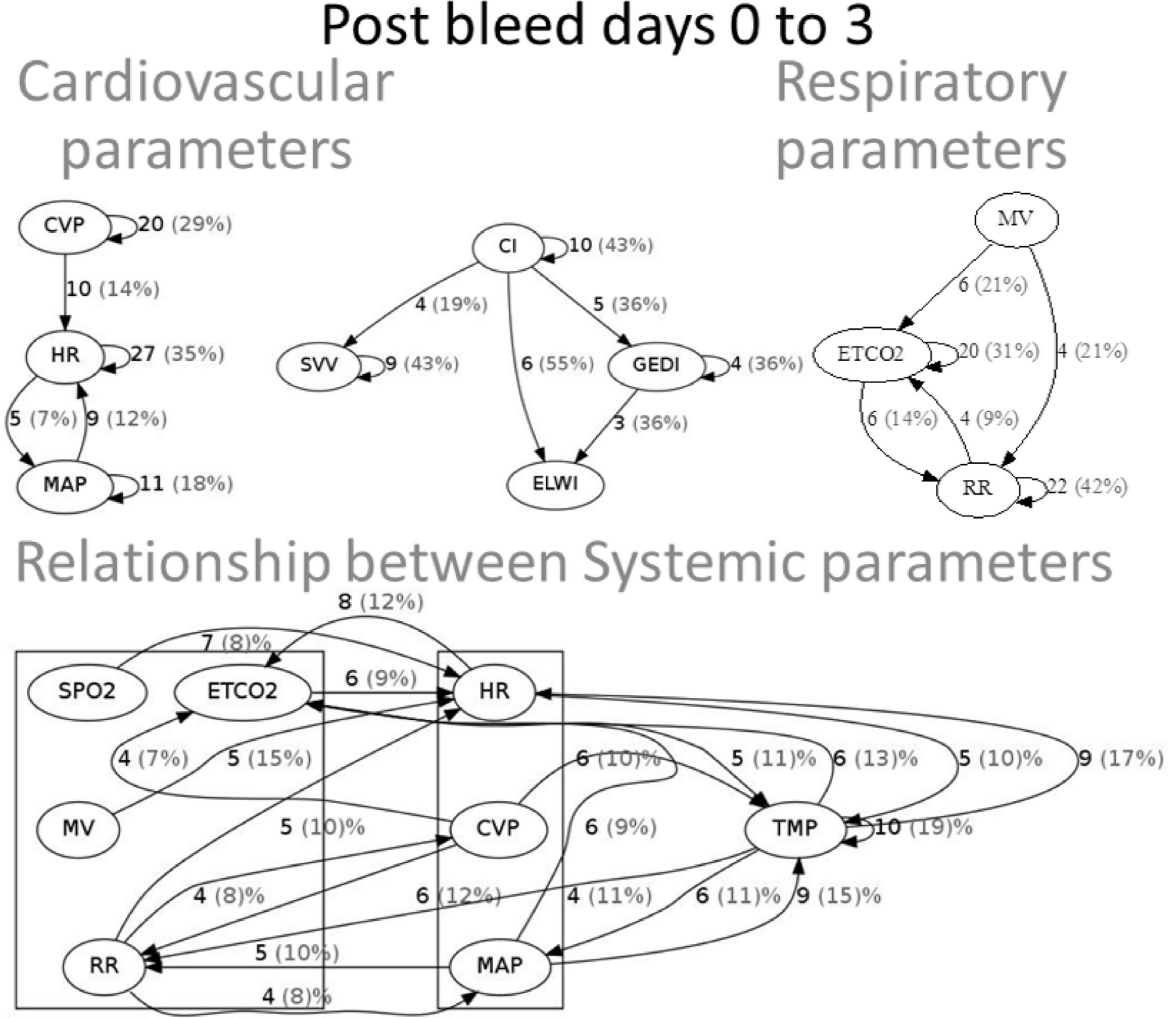
Causal relationships between systemic physiologic parameters during the initial post injury phase (days 0 to 3). Cardiovascular (top row, left panel), pulse contour analysis (top row, middle panel), respiratory (top row, right panel), and cardio-respiratory (bottom row) relationships.

**Fig 3:**
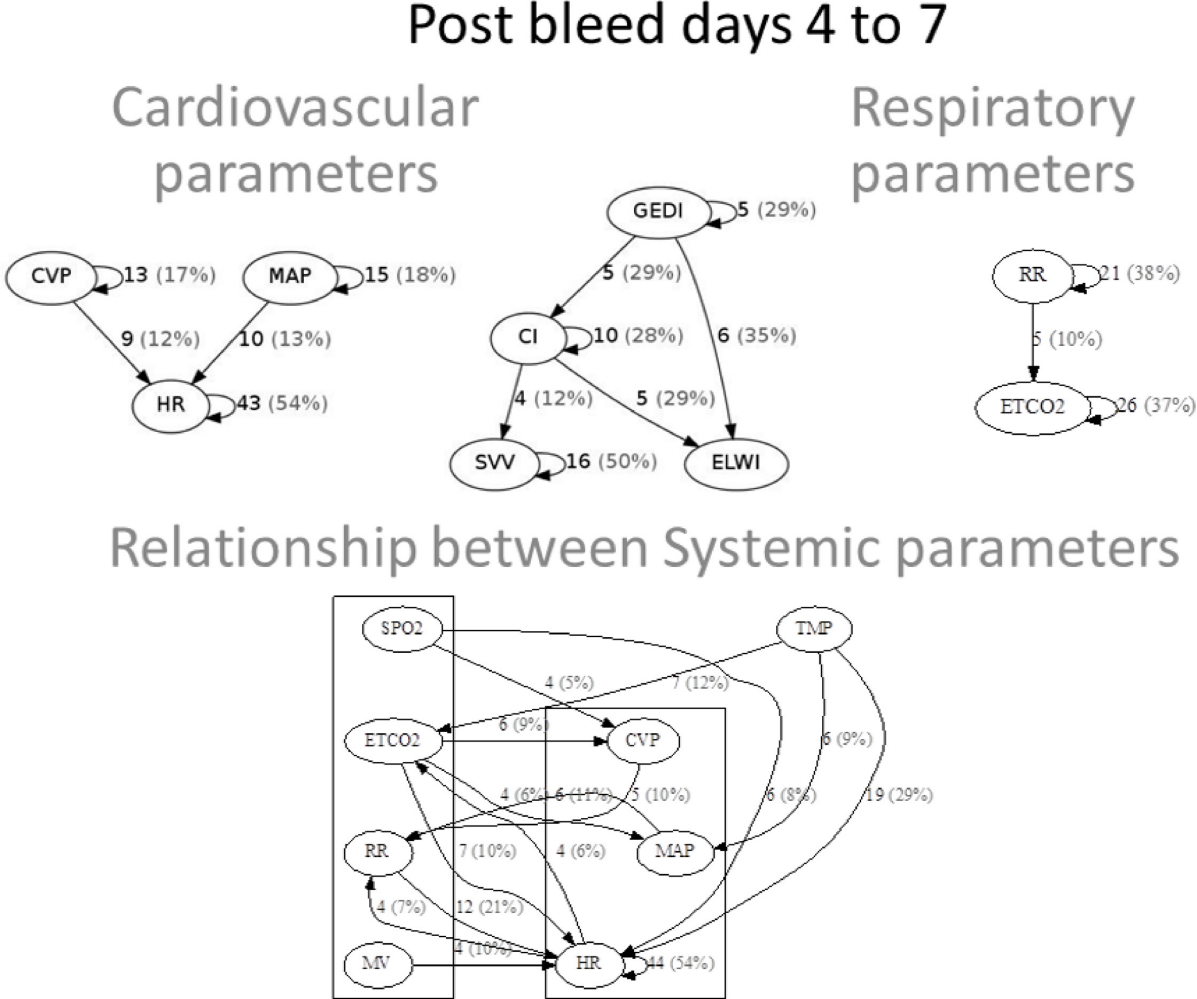
Causal relationships between systemic physiologic parameters during the second post injury phase (days 4 to 7). Cardiovascular (top row, left panel), pulse contour analysis (top row, middle panel), respiratory (top row, right panel), and cardio-respiratory (bottom row) relationships.

Systemic physiologic parameters (Fig 2). During the initial phase, we identified causal relationships between respiratory and cardiovascular parameters. Because not all measures were recorded for all patients, the number of patients that exhibited a specific causal relationship is indicated in the figures as both a frequency and a percent total of the number of patients that had the pair of variables measured. Pulse contour analysis is a measure derived from arterial waveform analysis and was only available in 11 to 21 patients depending on the variable in question. In this small subpopulation of patients the identified relationships were primarily self-referential (Fig 2 top row, middle panel). Among cardio-respiratory parameters collected on most patients, several bidirectional causal relationships were identified (Fig 2, bottom row) including mean arterial pressure (MAP) with respiratory rate (RR), and RR with central venous pressure (CVP). Unidirectional relationships identified were minute ventilation (MV) on heart rate (HR), RR on HR, and SPO2 on HR. Body temperature had causal relationships with a number of cardiorespiratory parameters including bidirectional relationships with endtidal carbon dioxide (ETCO2), HR, and MAP, as well as a unidirectional causal effect of temperature on RR and CVP on temperature. Overall most causal relationships within cardiovascular and respiratory parameters and the relationship between systemic parameters and temperature remained during the second phase. Interestingly, many more cardiorespiratory links were identified (Fig 3).

**Fig 4:**
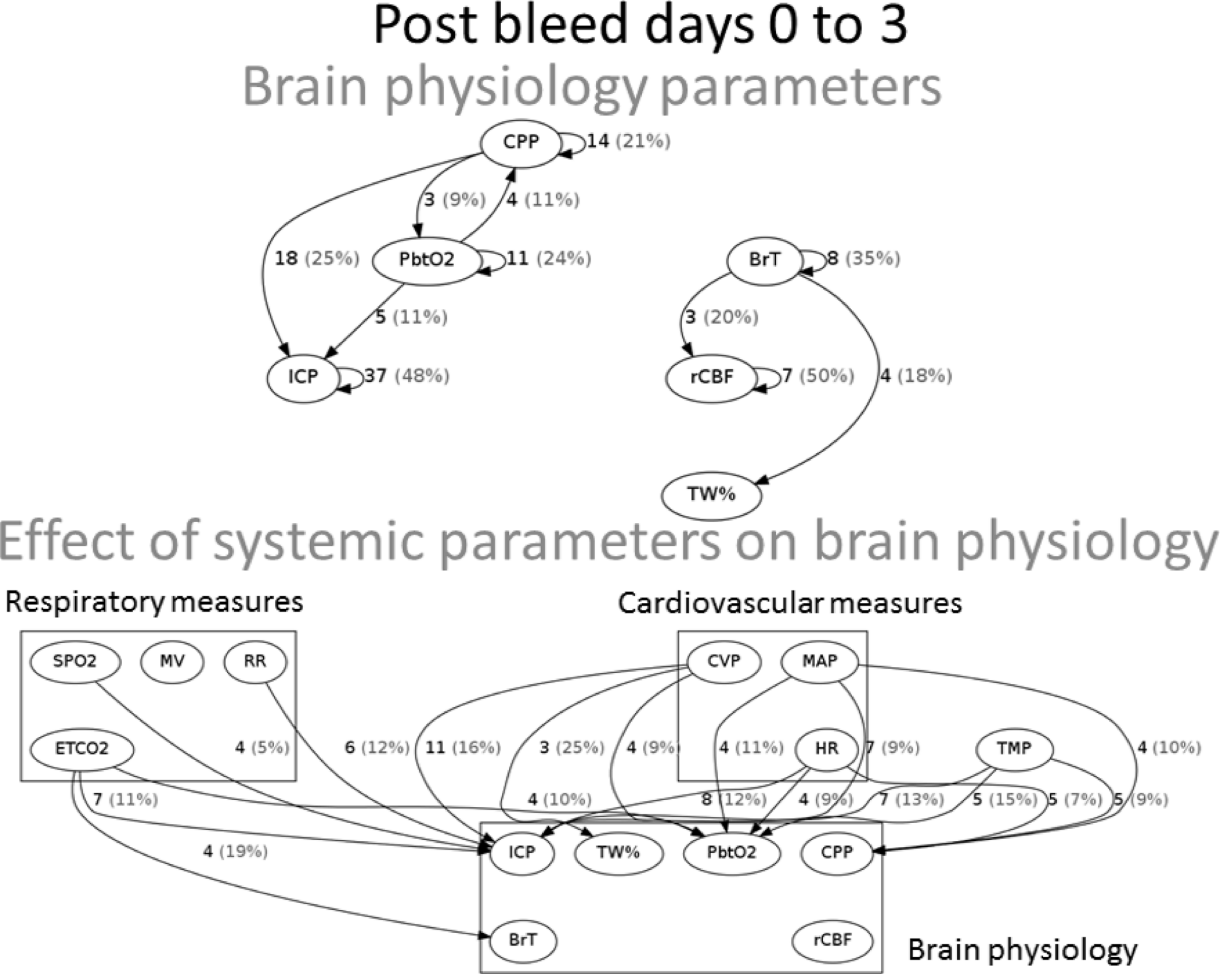
Brain physiology phase 1: Relationships within brain monitoring parameters (top row) and with systemic parameters (bottom row).

**Fig 5:**
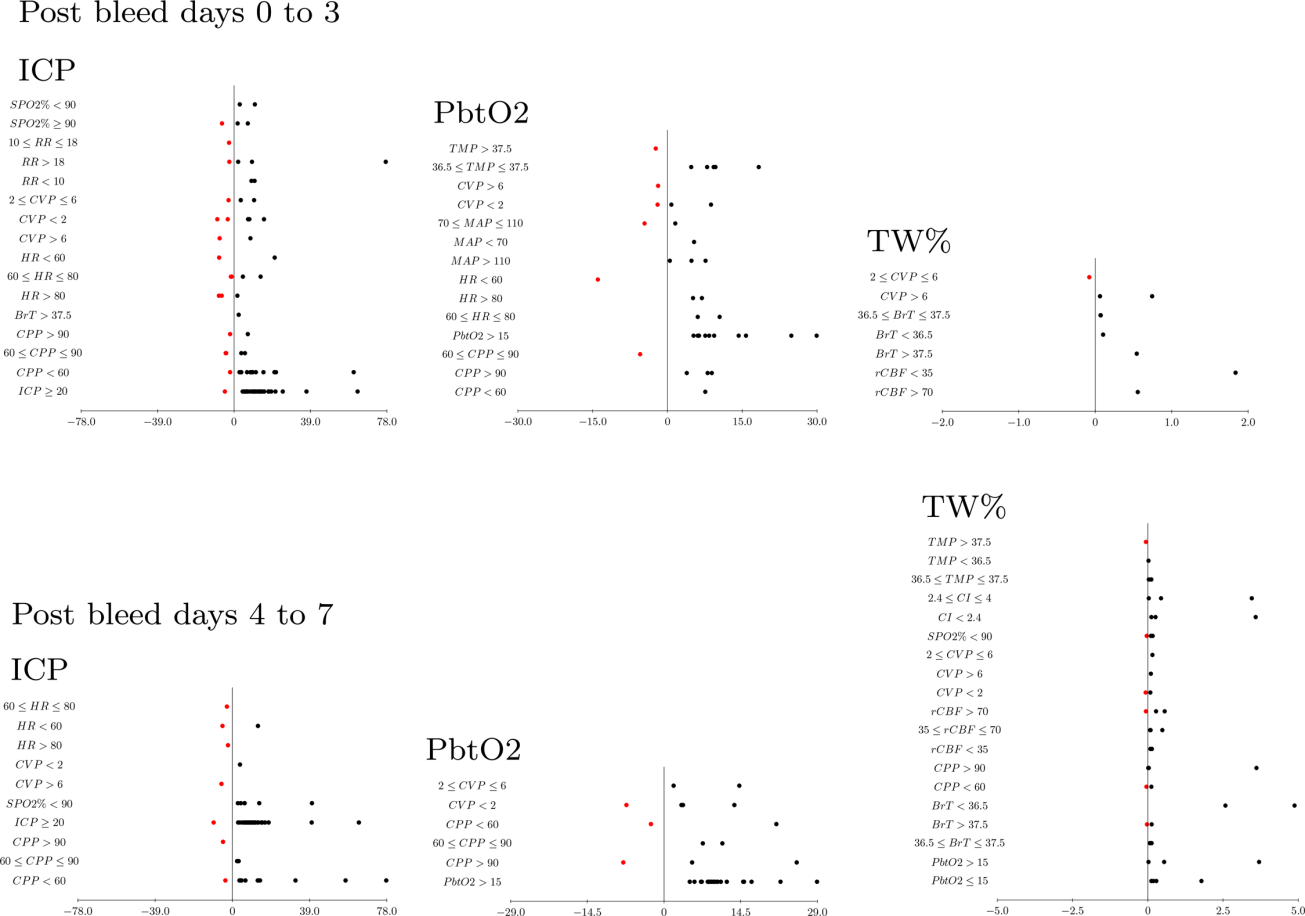
Directionality and effect size of causal inferences. Data is displayed for intracranial pressure (ICP), partial brain tissue oxygenation (pbtO2), and total brain water content (TW%; each dot signifies one patient; red dots indicated a decrease and a black dot an increase in the variable of interest).

**Fig 6:**
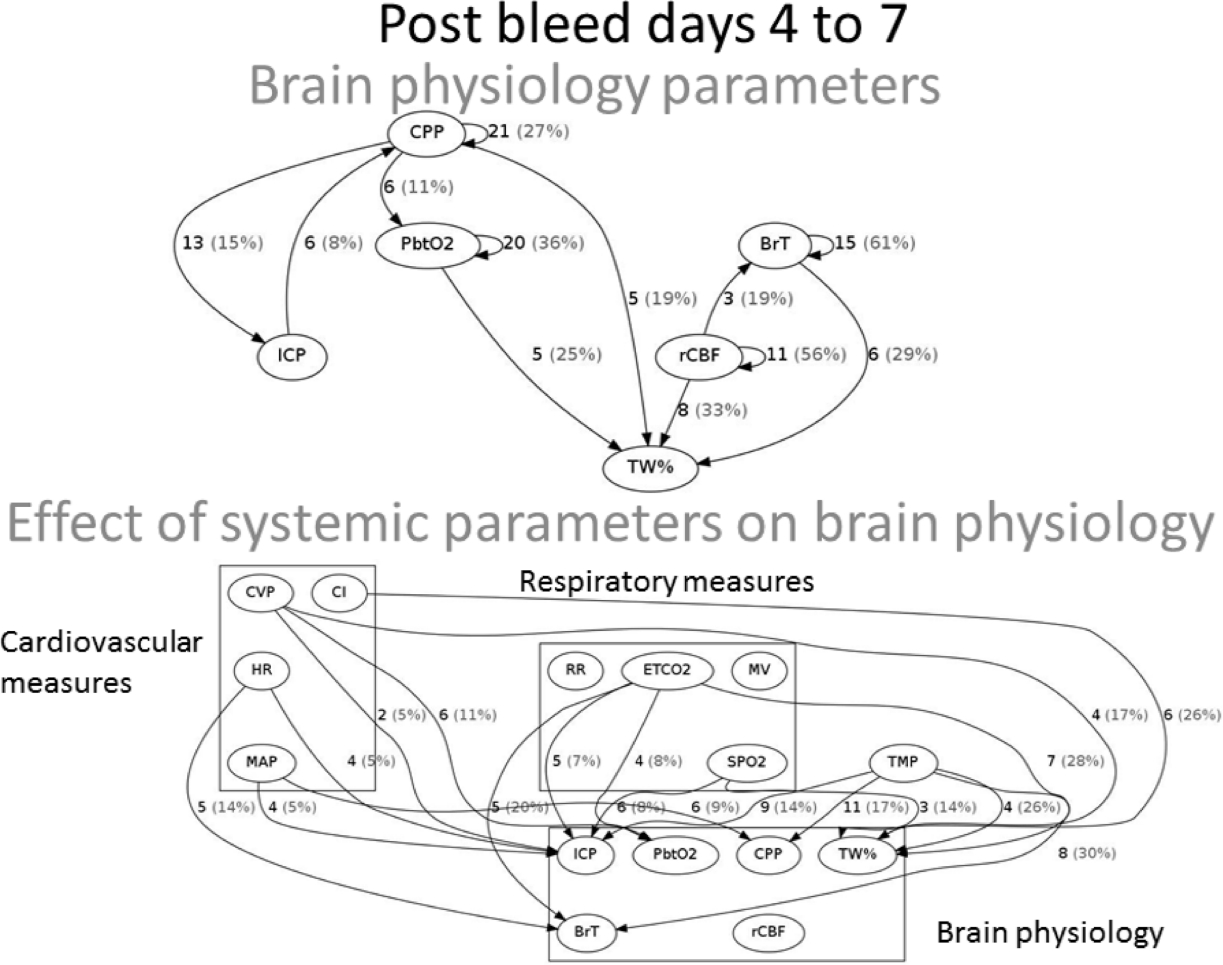
Brain physiology phase 2: Relationships within brain monitoring parameters (top row) and with systemic parameters (bottom row).

Brain physiology initial phase (post SAH day 0 to 3). **ICP.** A bidirectional causal relationship involving cerebral perfusion pressure (CPP) and pbtO2 was identified. Also causal effects were seen from pbtO2 and CPP on ICP (Fig 4). During the initial 96 hours after SAH, ICP was further influenced by cardiovascular (HR, CVP), respiratory parameters (ETCO2, RR, SPO2), and temperature. ICP did not causally influence any measures but was causally affected by pbtO2. The parameter most frequently causally linked to ICP elevation was a low CPP (Fig 5). **PbtO2.** Cardiovascular variables (MAP, HR, CVP), ETCO2, and temperature were found to causally affect pbtO2. CPP and pbtO2 had a bidirectional relationship and pbtO2 caused changes in ICP. Higher pbtO2 measures were most frequently linked to normothermia (compared to fever), and higher MAP and CPP measures (Fig 3). **Blood flow and water content.** BrT caused changes in TW% and rCBF. ETCO2 caused changes in BrT and rCBF caused changes in cardiac index (CI). Increasing TW% was primarily linked to parameters related to volume status (CVP, Fig 6).

Brain physiology secondary phase (post SAH day 4 to 7). 32% (N = 31) of patients developed brain ischemia, also known as delayed cerebral ischemia (DCI). **ICP**. Again, tightly linked relationships were seen between MAP, ICP, and CPP (Fig 6). Temperature, ETCO2, HR, CVP, and oxygen saturation were found to causally affect ICP, while ICP caused changes in HR. The most frequent increases in ICP were causally linked to low CPP and low oxygen saturations measures (Fig 5). **PbtO2**. CVP, ETCO2, and CPP were found to cause changes in pbtO2 (Fig 6), while pbtO2 caused changes in ETCO2, HR, and TW%. **Blood flow and water content**. The following variables caused changes in TW%: O2Sat, CVP, CI, CPP, temperature, ETCO2, pbtO2, BrT, and rCBF. Increasing TW% was primarily linked to parameters related to low brain oxygenation (Fig 5).

Phenotyping. The causal relationships inferred reflect the heterogeneity in the population, with most relationships being found in subsets rather than all patients and some variables having a positive impact in some patients and a negative impact in others. However, we did not identify any patient characteristics that differentiated these groups. To test this, we performed cluster analysis to distribute patients into distinct groups based on parameters that are known to affect hospital course, such as severity of disease, age, or development of cerebral edema, as well as outcome parameters. There was no consistent relationship between any of the clusters (or even averaged close clusters) with causal relationships. The mean distances between clusters representing the patients for any relationship were not smaller than expected from a random distribution.

## Discussion

We identified biologically plausible causal relationships using physiologic data routinely collected after brain injury. Combining our causal inference and imputation techniques enabled this real-world data to be used to gain insight into the physiology of patients recovering from hemorrhagic stroke, despite many missing values and a poor signal-to-noise ratio. Interestingly, we confirmed the complex dependencies of partial brain tissue oxygenation (pbtO2) measures primarily on cardiovascular and pressure measures. This complexity illustrates the challenges clinicians face in interpreting this signal at the bedside.[12] Brain water content, a novel measure for brain swelling,[20] appears to be initially dependent on fluid status but later also on brain oxygenation suggesting that the etiology of initial and secondary brain swelling may differ[28]. We did not identify demographic or disease specific patient phenotypes that differentiated those with from those without causal relationships, but found fewer relationships when data had low variability or high nonstationarity.

Methodology. One of the biggest challenges in analyzing real-world physiologic data is dealing with variables measured at different timescales and with large gaps between measurements. Many methods exist for imputing missing values, but these ignore the temporal dependencies of these data. Further, missing values for a variable can be dependent on the variable itself (e.g. when a single device malfunctions) or on other variables (e.g. if monitors are disconnected to perform an intervention) or both. These dependencies also exist across time, so the value of blood flow may depend on temperature with a delay, rather than instantaneously. To address this we developed an imputation method that combines the Fast Fourier Transform with a time-lagged K-Nearest Neighbors method (the combination is called FLk-NN) to account for both types of missingness. This also enables imputation for time instances where all data are missing (e.g. when a device measuring multiple variables is disconnected). The imputation method was validated on a subset of 9 patients from the full dataset. To simulate missingness, patients with more present values were used so that the amount of missingness could be synthetically varied by deleting varying percentages of data (while still having ground truth). In comparisons against leading imputation methods on these data, error rates were the lowest for all amounts of missing data and ranged from a mean absolute error (MAE) of 0.02 for 5% missing to 0.038 for 50% missing.[22] While imputation was successful even with gaps as long as an hour, we imposed the condition that no values were imputed for gaps over 30 minutes (so no values were imputed for microdialysis data, since these are spaced approximately an hour apart and we did not think imputation would be meaningful).

Causal inference. Relative consistency of the internal environment known as homeostasis is a fundamental prerequisite for biological functioning and governed by a series of complex interdependent control systems. This biological balance is maintained both by a network of interconnected physiologic properties with bidirectional dependencies between variables (i.e., neuronal activity, brain temperature, heart rate, and blood flow) as well as more direct corrective steps (i.e., vasodilatation in hypoperfused brain).[29] Following acute brain injury these autoregulatory processes are often impaired leading to dynamic alterations in the relationship between physiologic properties.[30] Much of our understanding of brain pathophysiology is based on static observations such as autopsy and structural imaging. This approach insufficiently replicates dynamic human pathophysiologic states, as illustrated by the recently debunked long held assumption that the brain can only metabolize glucose[31] indicating that brain metabolism during acute injury switches from glucose to lactate as its main substrate. The causal inference method applied here allows efficient inference of physiologic relationships and their timing.[32, 33] One of the key challenges of computational inference has been limited attention paid to the timing of relationships (ignored by Bayesian networks, and treated as discrete lags by Granger causality and dynamic Bayesian networks, DBNs). In clinical data, though, relationships occur at multiple timescales.

Given the significant missing data and noise, even if relationships did have a single discrete lag, we would not observe them as such. Instead, by using time windows (rather than lags) and enabling significance of each causal relationship to be assessed individually (rather than finding a network where one error or omission can have a cascading effect), we were able to efficiently find individualized physiological models. In particular, in preliminary tests of DBNs the methods either overfit to the noise in each patient’s data or make few discoveries, leading to little overlap in both cases.

Initial post injury phase. Relationships between physiologic parameters are expected to change over time following acute brain injury particularly in SAH as recovery from the initial injury and development of secondary complications follow a predictable but significantly variable time course. As monitoring only starts following the injury we are unable to provide a pre-injury baseline but most complications such as delayed cerebral ischemia and secondary brain swelling occur after the initial four post bleed days. As in prior analyses, [22,23] we therefore chose to split the dataset and separately analyze the initial 3 days and then the following post bleed days 4 to 7.

Systemic physiology. Causal inferences were identified within cardiovascular (MAP, HR, and CVP) and respiratory parameters (RR, MV, and ETCO2). These confirm widely accepted fundamental physiologic principles such as that seen between RR or MV and ETCO2. Our observations confirm that these relationships are robust in the setting of tightly controlled systemic physiology as management for these patients included intubation, respiratory management strategies to keep close ETCO2 goals, tight blood pressure control including vasopressors and antihypertensives as needed, and careful fluid management. These management strategies may in part also explain the identified cardio-respiratory relationships (MAP, HR, and CVP to RR, MV, oxygen saturation). However, direct vagal nerve mediated cardiorespiratory reflexes or common sympathetic or parasympathetic stimulation may also play a role. As expected, body temperature had a number of systemic, mostly bidirectional, causal links with respiratory (RR, ETCO2) and cardiovascular parameters (HR, and MAP). Temperature is likely linked to these physiologic parameters via the sympathetic and parasympathetic nervous system but treatment strategies such as targeted temperature management and medications given to counteract shivering are additional factors that may add to these causal links.

Invasive multimodality brain monitoring allows insights into dynamic brain physiology in pathologic states such as trauma or stroke. These monitors are increasingly used to directly measure intracranial pressure, brain oxygenation, cerebral blood flow, oxygen extraction, electrical activity, brain temperature, cerebral water content, and metabolites such as glucose, lactate, glycerol, glutamate, and pyruvate.[12, 13, 20] Current practice utilizes these measures mostly in a threshold based approach and does not take the complex interdependencies into account to guide treatment of elevated intracranial pressure, ischemia, and seizures.[34]

ICP. Confirming the widely accepted observation that MAP changes may directly lead to ICP changes particularly in patients with abnormal vasoreactivity following acute brain injury, a causal inference of MAP on ICP was seen. Similar mechanisms may explain the effect of other cardiovascular parameters on ICP (HR, CVP) but sympathetic stimulation or the Cushing’s reflex may also be a common shared link. As CPP is calculated from MAP and ICP, it is not surprising to see several bidirectional relationships between CPP and both MAP and ICP. Note that while CPP = MAP-ICP, we do not necessarily expect relationships between all three in all patients. That is because calculations of CPP reflect MAP and ICP at a single timepoint, and our causal inference method requires that causes must occur prior to their effects (rather than simultaneously), and it is not the case that later values of MAP and ICP must necessarily depend on earlier ones.

Respiratory parameters (ETCO2 and RR) directly link to cerebral vascular diameter via PCO2 or pH changes in the blood. Neuronal injury from systemic hypoxia (reflected in oxygen saturation) may lead to brain swelling and raised ICP. Several studies have associated elevated ICP with fever,[35] a relationship we were able to confirm. PbtO2 changes were related to changes in ICP, which could be explained based on a number of possible pathophysiologic processes including herniation, strokes, brain swelling, or hypoperfusion. Interestingly, no other effects of ICP were identified amongst systemic or brain physiologic parameters. In the second phase the majority of relationships remained stable but most importantly pbtO2 changes were no longer influenced by ICP changes. It is possible that ICP and pbtO2 were much better controlled during the second phase but variability and non-stationarity did not differ between the two time periods.

PBTO2. Several techniques have been introduced to generate estimates of brain oxygenation at the bedside utilizing either changes in the electrical properties detected at a Clark type electrode as in the current study or direct tissue oximetry using an optical system. PbtO2 is often used as a surrogate for blood flow changes in patients monitored for vasospasm following SAH or to guide blood pressure targets following TBI.[2] Our results support that pbtO2 is part of a complex integrated network of physiologic dependencies adding to the challenge of clinically interpreting measurements and changes in this parameter.[36] We found causal inferences for pbtO2 amongst both, factors affecting oxygen supply (MAP, HR, CVP, ETCO2) and oxygen demand of brain tissue (temperature). Additionally, causal inferences of pbtO2 on ICP and CPP were identified early in the disease course (days 0–3). This suggests that pbtO2 alterations may be linked to additional brain injury shortly after brain injury.

Brain water content. Diffuse brain swelling known as global cerebral edema (GCE) after SAH is found in 8% of patients on admission and 12% during hospital course and is associated with poor functional outcome.[28] The etiology of cerebral edema following SAH is not completely clear and a number of potential mechanisms have been proposed including perfusion changes,[37, 38] inflammation, hypoxia, and sodium dysregulation.[37, 39] Recently, a methodology to continuously measure total brain water content (TW%) as a correlate of GCE has been introduced.[20] Our results support the relationship between cerebral perfusion changes and GCE throughout the clinical course (effect of rCBF and CVP, Figs 2 and 4), supporting prior observations associating GCE with mediations given to raise the blood pressure.[28] Interestingly, in the second phase (day 5–8) effects related to oxygenation were also found to be related to GCE supporting the notion that mechanisms of admission GCE may differ from those that develop GCE during the hospital course.[28] A number of potential treatments have been proposed including agents that restore a leaky blood brain barrier such as aquaporins or steroids, medications to modify blood pressure, dehydrating agents such as mannitol or hypertonic solutions, or measures to alter regional cerebral blood flow such as vasodilators.[39]

Limitations. Physiologic relationships may continuously and gradually change making them difficult to detect. Our approach partly handles this change by studying causality in two different time periods separately, but it remains to discover the correct timings in a data driven way and most critically to discover how relationships may change gradually over time. We studied causal relationships that occurred over a time frame of up to one hour after generating one minute averages of each variable. We may therefore have missed faster (<1 minute) or delayed (>1 hour) causal relationships.

Causal inference for individuals further requires that variables have variation (so we cannot discover relationships between static factors such as sex and outcomes), and can only infer relationships between the variables that are observed. This limitation is illustrated by the lack of an identified causal relationship between SPO2 and pbtO2, which we would expect and may be caused by the minimal variability of SPO2 values. Additionally, if there is an unmeasured variable that causes two observed ones, we may identify a relationship between them if one follows the other with a consistent time lag. It is further important to realize that we observe the entire network in a situation of stress (i.e., acute brain injury) and that the detected relationships link upstream and downstream regulatory responses that are intended to restore this equilibrium. A major limitation for studying brain physiology in the pathologic states is the difficulty to differentiate between ongoing pathologic processes and adaptive, potentially protective, responses. This may require interventional trials or moving back and forth between bench and bedside.

Challenges of developing a more comprehensive understanding of brain physiology in humans include limitations of the available datasets and prior analytical approaches. Most datasets have a very limited set of physiologic parameters at a low time resolution. The chosen physiologic parameters in this study are only a selection of a convenience sample of variables chosen based on their increasing use in clinical practice. Others such as microdialysis (only collected hourly), imaging (very poor spatial resolution), and EEG (adding a large degree of complexity) were not included but may be explored in the future. Cerebral blood flow and TW% in this study were measured using a temperature dissipation methodology and not surprisingly we found that this was dependent on BrT. This likely represents a technique dependent relationship as both variables were measured with the same probe. For the chosen methodology we were required to categorize physiologic measures into ordinal scales of normal and abnormal, as well as if applicable into abnormally high and abnormally low values. These cut points were based on cut points in the literature and clinical judgment if these were not available (see Methods and Table 2). This needs to be mentioned as a potential limitation of the study as different cut points could potentially modify the causal inferences. It does however not alter the finding of those relationships that were identified in the dataset. Additionally, the primary point of this experiment was not to define physiologic thresholds as we do not have a ground truth but rather to delineate how deflections from the normal range relate to each other.
